# Rhabdomyolysis and Liver Failure Associated With Nontyphoidal Salmonella Infection: A Case Report

**DOI:** 10.7759/cureus.94622

**Published:** 2025-10-15

**Authors:** Armando Ugarte, Cameau Charleus, Smita Mathur, Samrawit W Zinabu, Jeffrey Palmer, Kevin Boluyt, Mateo Ugarte, Miriam Michael

**Affiliations:** 1 Internal Medicine, Howard University College of Medicine, Washington, DC, USA; 2 Internal Medicine, University of Maryland Medical Center, Baltimore, USA; 3 Internal Medicine, Howard University Hospital, Washington, DC, USA; 4 Internal Medicine, George Washington University School of Medicine and Health Sciences, Washington, DC, USA

**Keywords:** acute kidney injury, compartment syndrome, hyperthermia, rhabdomyolysis, salmonella

## Abstract

Rhabdomyolysis is a rare outcome of a *Salmonella *infection. This case presents a 21-year-old male with rhabdomyolysis likely caused by an ongoing *Salmonella *infection. He initially presented to the emergency department (ED) with severe hyperthermia following extraneous physical activity. His creatine phosphokinase (CPK) levels and other labs demonstrated evidence of rhabdomyolysis and acute kidney injury. His stool PCR was also positive for *Salmonella*. His clinical condition worsened with findings concerning bilateral compartment syndrome, so he underwent bilateral lower extremity fasciotomy. There are limited case reports of a *Salmonella *infection causing rhabdomyolysis and progressing to compartment syndrome. This case highlights the importance of expanding the causes of rhabdomyolysis beyond its typical causes, such as crush injury and strenuous exercise. A systemic approach leads to a more favorable outcome and prevents further progression or complications of rhabdomyolysis.

## Introduction

Rhabdomyolysis is a clinical syndrome of acute necrosis of skeletal muscle resulting in markedly elevated plasma creatine kinase (CK) levels and myoglobinuria [1]. Rhabdomyolysis causes symptoms such as myalgia, weakness, and dark, tea-colored urine [1]. Rhabdomyolysis has an array of potential causes, including crush injuries to muscle, prolonged immobility, medications, and *Salmonella *infection, although rare [1-6]. *Salmonella *remains one of the most common causes of gastrointestinal infection in the world and can cause two types of disease. Enteric fever is caused by *Salmonella typhi *and *Salmonella paratyphi*, while gastroenteritis is caused by other strains of *Salmonella*, known as nontyphoidal strains [2]. We report one case in which nontyphoidal *Salmonella *complicated a case of rhabdomyolysis, which later progressed to compartment syndrome, a rare outcome. Prompt diagnosis and treatment are crucial to prevent the progression of *Salmonella *and further complications of this infection. Given the absence of previously reported cases in the literature demonstrating the same degree of rhabdomyolysis and liver failure due to exercise and hyperthermia, we considered alternative explanations for the observed clinical presentation. *Salmonella *infection emerged as a potential culprit, as it has been associated with both rhabdomyolysis with compartment syndrome and hepatic dysfunction in some cases [1-6]. Systemic infections, including those caused by *Salmonella *species, can induce a severe inflammatory response, leading to muscle breakdown, metabolic disturbances, and multi-organ involvement [7-8].

## Case presentation

A 21-year-old male with no significant past medical history was admitted to the emergency department (ED) in August 2023 with severe hyperthermia (temperature: 106°F) following physical activity. The patient was an avid athlete who practiced martial arts twice a week and maintained a relatively healthy lifestyle. On this occasion, the patient was running outside when he suddenly collapsed to the ground and became altered. On admission, he was confused and agitated, requiring Lorazepam administration for sedation. Imaging ruled out an acute neurological cause and was unremarkable. Toxicology screens for alcohol and drugs of abuse also came back negative. He was treated with ice packs and cooling blankets, which successfully reduced his temperature. Given the severity of his presentation, he was closely monitored in the intensive care unit.

Laboratory tests on admission revealed severe rhabdomyolysis, with severely elevated creatine phosphokinase (CPK) and creatinine levels, indicating acute kidney injury (Table 1). He was started on aggressive intravenous hydration, but despite this, his creatinine increased the following day (Table 1). A stool PCR panel returned positive for *Salmonella enterica* and *bongori *DNA, supporting an infectious etiology of his rhabdomyolysis and systemic complications.

**Table 1:** Lab values throughout the patient’s hospital course, illustrating the acute renal and hepatic dysfunction on initial presentation Reference ranges were determined using values from the American Board of Internal Medicine. ALT = alanine aminotransferase; AST = aspartate aminotransferase; BUN = blood urea nitrogen; CPK = creatine phosphokinase; eGFR = estimated glomerular filtration rate

Hospital Day	1	2	3	20	27	Reference Range
Creatinine (mg/dL)	2.58	2.32	1.89	3.64	2.78	0.70-1.30
CPK (U/L)	320,000	257,000	102,000	16,000	3,300	55-170
Myoglobin (mmol/L)	91,740	78, 450	43, 800	11,000	5,600	<100
BUN (mg/dL)	22	18	15	60	75	8-20
eGFR (mL/min/1.73m^2^)	35	40	51	23	32	90-120
Serum Glucose (mg/dL)	69	40	109	110	103	70-99
Total Protein (g/dL)	5.1	5	3.9	5.2	6	6.0-8.3
Serum Albumin (g/dL)	2.5	2.6	1.8	2.2	2.6	3.5-5.5
AST (U/L)	6,073	7,885	5,828	1,371	109	10-40
ALT (U/L)	2,916	4,375	3,954	431	179	10-40
Bilirubin Total (mg/dL)	3.1	4.2	4.3	1.7	0.4	0.3-1.0
Bilirubin Direct (mg/dL)	1.8	2.6	3.4	1.4	0.1	0.1-0.3
Lactate (mg/dL)	4.1	5.4	3.7	1.5	1.1	0.7-2.1
Calcium (mg/dL)	6.7	6.8	6.7	7.0	8.6	8.6-10.2
Phosphorus (mg/dL)	5.1	4.7	3.4	4.8	4.5	3.0-4.5

**Figure 1:**
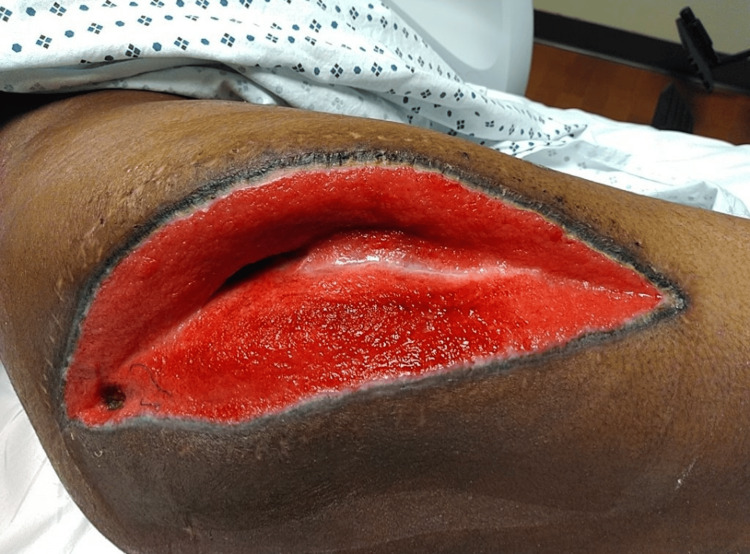
Right leg fasciotomy

**Figure 2:**
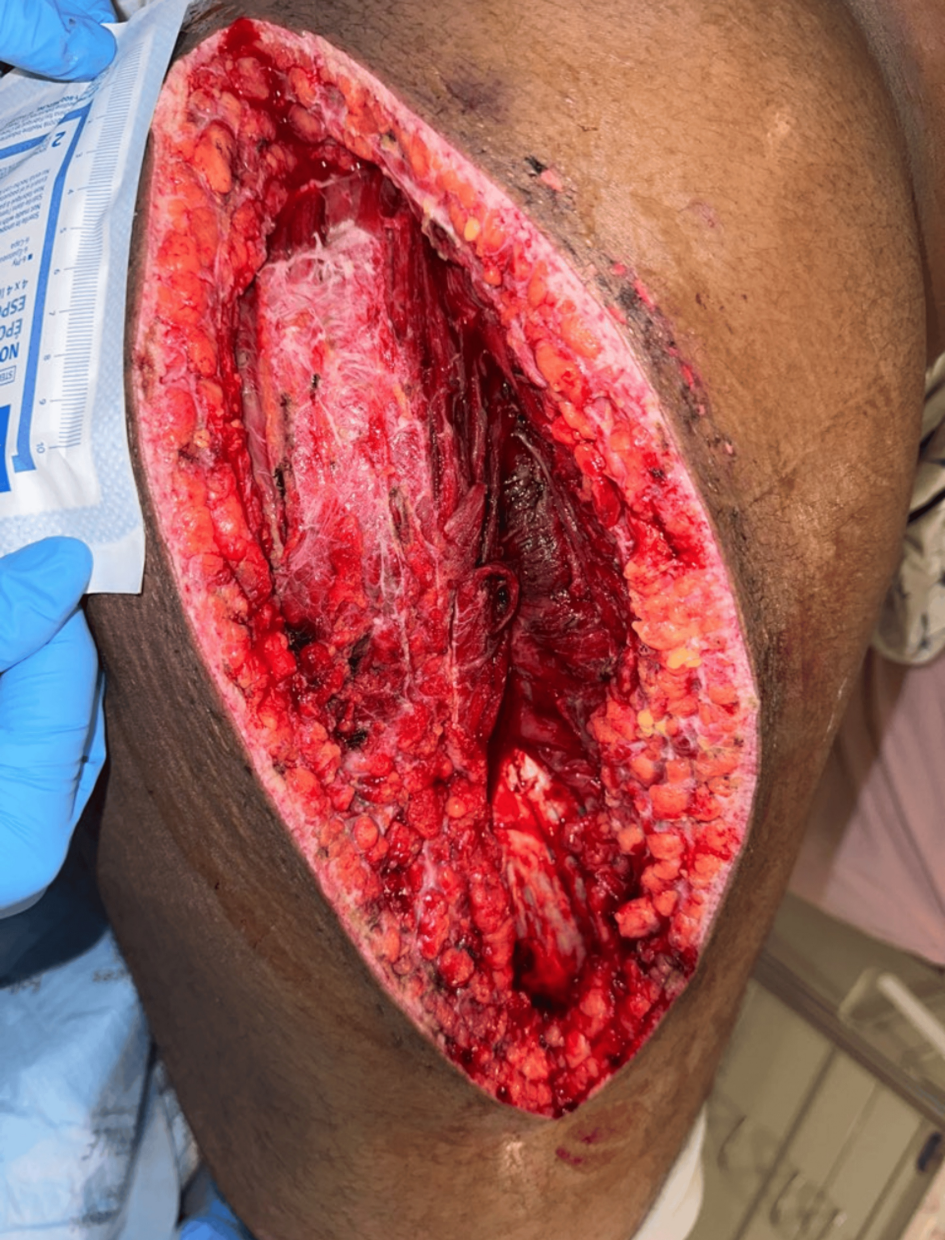
Reopening of right leg fasciotomy

The patient developed new-onset edema and severe pain, raising concern for bilateral compartment syndrome. He underwent an emergency bilateral lower extremity fasciotomy and was transferred to a tertiary shock and trauma center (Figure 1). Due to concerns of worsening compartment syndrome, the right fasciotomy was reopened, but the muscle was viable (Figure 2). The left fasciotomy remained open, and he was transferred to the medical ICU with a wound VAC system. Despite treatment, CK and myoglobin levels remained elevated, necessitating a second right fasciotomy, reopening 20 days later. Over time, renal function improved, transitioning into the polyuric phase. Nephrology managed his fluids, replacing 50% of urine output with half-normal saline to prevent hypotension and dehydration. By hospital day 27, myoglobin and CPK decreased significantly, showing a great improvement in this patient’s renal function (Table 1).

On admission, the patient also developed acute liver failure with an INR of 3.2. The patient required molecular adsorbent recirculating system (MARS) therapy from hospital day 2 to day 7. He was monitored closely by gastroenterology and the liver transplant team. As his liver function steadily improved, further intervention was deemed unnecessary. The patient made a full recovery and was discharged from the hospital in September 2023, approximately one month after his admission.

## Discussion

Rhabdomyolysis is a rare but serious condition that can result from various causes, including trauma, extreme physical exertion, and systemic infections. Its pathogenesis is well-documented, with the final common pathway involving an excessive intracellular accumulation of calcium, leading to muscle cell membrane damage and necrosis [9]. When muscle injury or ATP depletion occurs, there is an influx of sodium (Na⁺) and calcium (Ca²⁺) ions into the cell, causing sustained myofibrillar contraction and further ATP depletion [9]. Elevated intracellular calcium disrupts membrane integrity, leading to the release of muscle contents, including creatine kinase (CK) and myoglobin, into the bloodstream, ultimately contributing to inflammatory muscle necrosis [9].

Infectious causes account for only 5% of rhabdomyolysis cases, with common bacterial pathogens including Streptococcus and Legionella, and viral etiologies such as influenza and HIV [10]. *Salmonella* is a particularly rare cause of rhabdomyolysis, making this case unique. Even more uncommon is its progression to compartment syndrome, which occurs in only 4% of rhabdomyolysis cases [11]. The combination of *Salmonella* infection, hyperthermia, and exercise-induced muscle stress likely contributed to the severity of muscle damage in this patient.

*Salmonella* infections primarily affect the gastrointestinal system, but extraintestinal complications such as myositis and rhabdomyolysis have been documented. A review of 60 cases of infection-associated rhabdomyolysis found that *Salmonella* was implicated in 10% of cases. However, among those with acute kidney injury (AKI), *Salmonella* accounted for 67%, a significantly higher rate than the overall AKI incidence of 57% [12].

The pathophysiology of *Salmonella*-induced rhabdomyolysis is thought to involve a combination of direct bacterial invasion of muscle tissue, toxin release leading to muscle necrosis, sepsis-induced tissue hypoxia and metabolic derangements, and severe dehydration with electrolyte imbalances, all of which contribute to muscle damage [13]. Additionally, *Salmonella*’s type III secretion system (T3SS) plays a crucial role in host cell invasion and cytoskeletal remodeling [14]. The bacterium injects effector proteins, including SipA and SipC, into host cells, promoting actin polymerization and membrane ruffling, which facilitates bacterial internalization [14]. This mechanism may also contribute to muscle tissue involvement, worsening inflammatory damage, and increasing the risk of compartment syndrome in enclosed muscle groups, such as the lower extremities in this patient. 

In this case, liver failure may be linked to both *Salmonella* infection and rhabdomyolysis through several mechanisms. *Salmonella* can cause a systemic inflammatory response, leading to sepsis and multi-organ dysfunction, including hepatocellular injury due to direct bacterial invasion or toxin-mediated damage. Additionally, rhabdomyolysis itself can contribute to liver failure through the release of massive amounts of myoglobin, which can overwhelm hepatic processing and lead to oxidative stress and hepatocyte injury. The combination of systemic infection, inflammation, and muscle breakdown likely exacerbated hepatic dysfunction, leading to liver failure. Furthermore, acute kidney injury secondary to rhabdomyolysis may have impaired the clearance of inflammatory mediators and toxins, further worsening liver injury. This case underscores the complex interplay between severe infection, muscle injury, and organ dysfunction, emphasizing the importance of early recognition and aggressive management to prevent fatal complications.

## Conclusions

This case highlights a rare but clinically significant association between nontyphoidal *Salmonella*, rhabdomyolysis, and compartment syndrome. While rhabdomyolysis is commonly linked to trauma, exertion, or viral infections, this patient’s presentation underscores the importance of considering infectious etiologies in patients presenting with severe rhabdomyolysis and liver dysfunction, particularly when traditional risk factors such as extreme exertion and heat exposure do not fully explain the severity of the clinical presentation. Early recognition and aggressive management are critical in preventing severe complications, particularly in cases where an atypical infectious trigger is suspected. This case expands the understanding of *Salmonella*’s extraintestinal manifestations, emphasizing the need for vigilance in identifying and managing rare presentations of rhabdomyolysis.

In addition, this case underscores the importance of multidisciplinary management when rhabdomyolysis presents with multi-organ involvement. Collaboration between infectious disease, nephrology, surgery, and hepatology was essential to optimize outcomes in this patient, reflecting the complex pathophysiology of *Salmonella*-induced systemic illness. Given the potential for *Salmonella *to cause extraintestinal manifestations such as rhabdomyolysis, compartment syndrome, and hepatic dysfunction, clinicians should maintain a high index of suspicion in patients presenting with severe systemic illness and positive stool studies. Future research is needed to better characterize the mechanisms by which *Salmonella *precipitates muscle and hepatic injury, as well as to determine optimal treatment strategies to mitigate morbidity. Increased recognition and reporting of such rare cases can improve understanding and guide management of similar presentations in the future.
